# Commentary: Perspectives on ADHD in children and adolescents as a social construct amidst rising prevalence of diagnosis and medication use

**DOI:** 10.3389/fpsyt.2024.1383492

**Published:** 2024-03-25

**Authors:** Tycho J. Dekkers

**Affiliations:** ^1^ Accare Child Study Center, Groningen, Netherlands; ^2^ Department of Psychiatry, University Medical Center Groningen (UMCG), Groningen, Netherlands; ^3^ Specialists in Youth and Family Care, Levvel, Amsterdam, Netherlands; ^4^ Department of Psychology, University of Amsterdam, Amsterdam, Netherlands; ^5^ Department of Child and Adolescent Psychiatry, Amsterdam University Medical Centers (AUMC), Amsterdam, Netherlands

**Keywords:** ADHD, decontextualization, stigma, medication, prognostic pessimism, paradigm

## 1 Introduction

The recent perspective article by Banaschewski et al. (1) in *Frontiers in Psychiatry* speculates about a potential “transformative paradigm shift” in the field of ADHD. This proposed paradigm shift centralizes around the notion that ADHD would not reflect a natural entity, but instead is best described as a social or cultural construct. I wholeheartedly agree with this statement, and in this commentary, I advocate for the necessity of this paradigm shift. It will yield significant benefits for individuals with ADHD in at least two crucial aspects: (1) by mitigating many of the negative consequences of receiving an ADHD classification, among which is an overreliance on medication, and (2) by improving the quality of our support.

## 2 Negative consequences of the current ADHD paradigm

The ADHD paradigm of the last decades, in which ADHD is mostly regarded as a natural entity, may have led to several unintended but potentially harmful consequences. Although everyone agrees that ADHD is the result of a complex interplay of biological and contextual factors (2), the current scientific and societal narrative predominantly focuses on biological factors such as genetics and brain functioning (3–7). Contextual factors like poverty (8), parental psychopathology (9), trauma (10), screentime (11), early deprivation (12), and being youngest in class (13) receive substantially less attention. The risk of this predominant emphasis on biological factors is that it may create a deterministic, individualized, and decontextualized view on ADHD. Decontextualization refers to the belief by children, their parents, teachers and clinicians that children themselves, or their brains, are primarily responsible for their symptoms. Decontextualization can have many negative consequences. Here, I will briefly discuss three: prognostic pessimism, stigma, and overreliance on medication.

### 2.1 Prognostic pessimism

First, decontextualization could lead to prognostic pessimism (i.e., having less hope for and lower expectations about the future). This is plausible as biological, decontextualized explanations of problem behaviors may imply that these problems are persistent and incurable (14–17). This phenomenon is firmly established for internalizing disorders: The more people attribute their problems to biology, the longer they expect them to last (18, 19), the poorer they perceive their own coping skills (20), and the more negative they rate their prognosis (21). A single study on ADHD mirrored this trend: experimentally induced individualized, decontextualized beliefs about ADHD led to pessimistic expectations about the child’s potential (22). This resembles with other work demonstrating that academic expectations of children with ADHD are disproportionately low (23), as also mentioned by Banaschewski and colleagues. This is crucial because expectations often become self-fulfilling prophecies (i.e., Pygmalion vs Golem effects; 24).

### 2.2 Stigma

Second, decontextualization could lead to stigmatization. Although not directly studied for ADHD, people are more reluctant to interact with people with mental health problems and perceive them as more dangerous, when they assume these mental health problems are caused by biological factors (17, 25, 26). Strikingly, this applies to clinicians as well: Clinicians linking their clients’ problems to biology display less empathy than clinicians attributing problems to non-biological factors (27).

### 2.3 Overreliance on medication

Third, decontextualization could lead to an overreliance on medication. When the starting point is that ADHD is a (neuro)biological disorder, clinical decision making may guide towards brain-focused solutions like medication (28, 29). Indeed, the more clinicians explain problems by biological factors, the higher they estimate the effectiveness of medication (30–33). Similarly, greater emphasis on biological problem explanations also correlates with reduced confidence in the effectiveness of non-pharmacological, often context-focused, treatments (19, 27, 29).

## 3 A paradigm shift to inform better support for children with ADHD

A shift towards a paradigm that more explicitly acknowledges the influence of contextual factors on ADHD will inform better support for children with ADHD. Clinical guidelines recommend both behavioral parent training and medication as first-choice interventions but in practice, many more children with ADHD receive medication than behavioral parent training (34). An important reason for this discrepancy is that a decontextualized view on ADHD guides children, parents, teachers and also clinicians towards medication *before* initiating other treatments. Instead, the notion of a socially constructed concept of ADHD, as proposed by Banaschewski and colleagues, automatically puts more emphasis on contextual factors. The logical consequence of this paradigm shift will hopefully be an increase in the implementation of context-focused behavioral interventions for children in ADHD.

Of these interventions, the evidence-base for behavioral parent training is most compelling (for meta-analyses, see 35–37). Treatment sequencing studies demonstrate that initiating treatment with such behavioral interventions yields superior outcomes compared to initiating treatment with medication, both in terms of effectiveness and costs (38, 39). Crucially, initiating treatment with behavioral interventions substantially reduces the need for medication later (40). By implementing such a stepped-care approach, overtreatment with medication is prevented while maintaining pharmacological treatment for those children who really need it.

## 4 Discussion

The upcoming paradigm shift in the field of ADHD will indeed be transformative, as anticipated by Banaschewski and colleagues. While it will undoubtedly come with many challenges, I see at least two clear gains: it will mitigate several unintended but harmful consequences of the current paradigm and it will pave the way to more balanced stepped-care recommendations, ultimately benefiting many children with ADHD and their families.

## Author contributions

TD: Writing – review & editing, Writing – original draft, Conceptualization.
